# For the Future Studies of Kaposi’s Sarcoma-Associated Herpesvirus

**DOI:** 10.3389/fmicb.2012.00237

**Published:** 2012-07-11

**Authors:** Keiji Ueda

**Affiliations:** ^1^Division of Virology, Department of Microbiology and Immunology, Osaka University Graduate School of MedicineOsaka, Japan

It is 18 years since Kaposi’s sarcoma-associated virus (KSHV), also called human herpesvirus 8 (HHV-8), was found from Kaposis’ sarcoma (KS) by Chang et al. (1994). More than 8, 000 reports have been published so far and we have learned many things from this virus. I would like to say it is about time to look back previous studies and to think what to study next on the virus, and planed a topic to think what to study next on the virus for future.

Herpesviruses have relatively big genomes and encode a 100 genes or so. Thus, the virion assembly/structure, gene expression regulation and attachment/entry are complicated and have known only an iceberg of them. Studying the details how the viruses run their life cycles and cause diseases in their processes will lead to exploring new therapeutic drugs/methods.

A viral life cycle starts from attachment on the susceptible cells and then, entry into the cells, followed by the viral gene expression, the genome replication, the particle assembly and finally the daughter viruses egress out of the cells. This process is skillfully built and all the viral genes are required for the process, though there are essential genes and non-essential ones. Viral pathogenesis could be established during this process by the interaction between viruses and host cells, and individual host systems such as immune system. In this topic, although I would like to cover all the processes, thankfully, 15 specialists in each field have contributed for this topic.

Polizzotto et al. (2012) described clinical manifestations of KSHV-associated diseases. So far, there were few reports on clinical manifestations of primary KHSV infection. In this term, KSHV inflammatory cytokine syndrome (KICS) is a new concept and we might have been looked over an important disease sign on KHSV infection. We will have to be more careful about what happens in primary KSHV infection than before.

Fukumoto et al. (2011) describe KSHV infection from a pathologist’s points of view. Pathologic study is very important to know what happens in the lesions. Currently, we are able to know what is going on only in the KSHV associated lesions such as Kaposi’s sarcoma, multicentric Castleman’s disease and primary effusion lymphomas (PEL) of human samples suffered from KSHV infection, but once an infection model is established, chronological pathologic studies will provide a lot of information on how KSHV-associated diseases are formed.

Chakraborty et al. (2012) review the entry mechanism of KSHV into cells. In general, herpesviruses can infect various kinds of cells *in vitro* including non-human cell lines, but the infectivity to B lymphocyte originated cells is very inefficient. Their report will give us a hint why such phenomenon happens.

An immediate early gene, *RTA* (reactivation and transcription activator) is very important for the viral lytic replication induction and shows multifunctions. We still have not understood how the factor functions. Guito and Lukac (2012) and Tsai et al. (2012) review or report mechanistic regulation of this strong transactivator, respectively.

Jackson et al. (2012) describe ORF57, which is also an interesting and multifunctional protein. This is involved in post-translational processes of the viral gene expression as sumoylation and ubiquitination described by Campbell and Izumiya (2012) and Ashizawa et al. (2012) respectively. We had believed that K-bZIP, a homolog of Epstein-Barr virus Zta was a transactivator and origin recognition factor in the lytic replication. K-bZIP, however, has other important roles for KSHV lytic replication. In latency, metabolism of LANA (latency-associated nuclear antigen) could be critical for KSHV-induced tumor formation and/or its phenotype.

Viral particle assembly is virologically an exciting and interesting field. There have been few reports on this, Sathish et al. (2012) try to search this issue.

The detail replication mechanism of KSHV in both lytic and latent phase has been still unclear. In latency, the virus is supposed to utilize host replication machinery including pre-replication complexes (pre-RC) for the viral replication initiation in the presence of LANA. The viral factor, LANA, is an essential factor, but its necessity has not been elucidated well. LANA binds with LANA-biding sites (LBS) and recruits origin recognition complexes (ORCs) on the viral replication origin (ori-P), which cannot account for necessity of the GC-rich element followed by LBS. Ohsaki and Ueda (2012) will give us a hint about this question.

Viral immune evasion system is very tactic to maintain its latency in case of herpesviruses. The maintenance of latency is then critical for the virus to wait for reactivation to produce daughter viruses, whose transition may a step for the viral oncogenic process. Lee et al. (2012) summarize KSHV immune evasion strategy and make a comment on the future landscape.

Kaposi’s sarcoma-associated virus mediated tumorigenesis including PEL and KS has been still unclear, though there are many reports on individual viral putative oncogenes. KSHV has not been reported to infect and immortalize and/or transform endothelial cells or peripheral blood mononuclear cells *in vitro*. And thus, we have not known how the viral genes with oncogenic potentials such as *vFLIP*, *vCYC*, *vGPCR* and so on in addition to *LANA* cooperate in the viral oncogenic process. DiMaio and Lagunoff (2012) address on this issue and look forward for this field.

MicroRNA is one of the hottest research fields even in KSHV. This kind of small RNA molecule seems to have profound effects on cellular processes and then viral activities but their details have not been elucidated totally. KSHV lytic and latent phases are regulated by viral but also cellular microRNAs. Two specialists; Liang et al. (2011) and Gottwein (2012) reveals the microRNA world of KSHV.

And finally, we have to think about treatment of KSHV-associated tumors such as KS, PEL and a lympho-proliferative disease, multicentric Castleman’s disease. It should be very hard to treat these tumors in the immunodeficient setting. It will be desirable if KSHV specific strategy is designed, since these tumors are very tightly linked with KSHV infection. Dittmer et al. (2012) contribute for this theme and discuss about it.
